# Intra-clutch and inter-colony variability in element concentrations in eggshells of the black-headed gull, *Chroicocephalus ridibundus*, in northern Poland

**DOI:** 10.1007/s11356-017-8635-z

**Published:** 2017-03-08

**Authors:** Ignacy Kitowski, Piotr Indykiewicz, Dariusz Wiącek, Dariusz Jakubas

**Affiliations:** 1 State School of Higher Education in Chełm, Pocztowa 54, 22-100 Chełm, Poland; 2 Department of Zoology and Landscaping, University of Sciences and Technology, Kordeckiego 20, 85-225 Bydgoszcz, Poland; 3 Institute of Agrophysics, Polish Academy of Sciences, Doświadczalna 4, 20-290 Lublin, Poland; 4 Department of Vertebrate Ecology and Zoology, University of Gdańsk, Wita Stwosza 59, 80-308 Gdańsk, Poland

**Keywords:** Eggshells, Heavy metals, Waterbirds, Contamination

## Abstract

Eggshells are good bioindicators of environmental contamination, and therefore, the concentrations of 17 trace elements in 87 eggshells of black-headed gulls, *Chroicocephalus ridibundus*, were determined in five breeding colonies in an area dominated by farmland in northern Poland. The intra-clutch variability in the eggshell concentrations of heavy metals and other elements was also investigated, and the concentrations of the elements showed the following pattern: Ca > Mg > Sr > Fe > Zn > Al > Cr > Se > Mn > Cu > Pb > As > Ni > Mo = V > Sc > Cd. The concentrations of Fe, Al, and Mn decreased with the order in which the eggs were laid, but Sr concentrations increased. In contrast, the concentration of Cu significantly increased with the laying date. The concentrations of all elements significantly differed among the studied colonies; the highest concentration of eight elements was found in the eggshells from the Kusowo colony, which may have resulted from the intensive use of fertilizers, manure, and slurry in the surrounding agricultural region. The concentrations of Mg, Sr, and Zn in the eggshells from Skoki Duże were higher than those of the other studied colonies, which may have occurred because the gulls were nesting in a functioning gravel pit; soil and the parent rock are natural reservoirs of these elements. The observed element levels indicate that the environment where the black-headed gull eggs were formed, i.e., primarily near the breeding colonies, remains in a relatively unpolluted state, which was reflected by the low levels of Cd, Ni, and Pb and the lack of measurable levels of Hg.

**Electronic supplementary material:**

The online version of this article (doi:10.1007/s11356-017-8635-z) contains supplementary material, which is available to authorized users.

## Introduction

Waterbirds are useful bioindicators because they are widely distributed, abundant, long-lived, and sensitive to environmental changes. Because they are also high in the food chain, they are suitable subjects for monitoring element bioaccumulation in tissues, including the liver, kidneys, and blood as well as bones and feathers (e.g., Horai et al. 2007; Kim and Oh 2012; Raygoza-Viera et al. 2013). Additionally, bird eggs are considered to be good bioindicators in long-term studies of geographic and temporal trends in environmental contamination as they may reflect the exposure of females to local trace elements (Burger 1994, 2002), which can be eliminated by sequestration in eggshells (Lam et al. 2005). The concentrations of elements in eggshells accurately indicate the levels in the environment and are especially positively related to the levels of metals in the blood and other tissues as well as the whole egg (Dauwe et al. 2005). There are some limitations in the use of eggshells for biomonitoring as the diets of some species may differ between the sexes (e.g., Forero et al. 2005), and therefore, the level of contamination may only be applicable to females. Furthermore, the eggshell signal only reflects a short period of time (pre-laying), but the spatial scale may vary depending on the strategy employed by the species to gain nutrients for egg production. Capital breeders store nutrients before breeding, while income breeders obtain nutrients daily during the pre-laying period (Reynolds 1997; Stephens et al. 2009). Thus, in income breeders, the trace elements in eggs reflect the contamination of the local breeding grounds, but in capital breeders, the trace elements indicate contamination in the wintering areas or stopover sites during the spring migration. Moreover, some elements are primarily accumulated in particular organs (e.g., Fe in the liver: Badzinski et al. 2009; Ba in the eyes: Nam et al. 2005; and Sr in bones: Skoric et al. 2012), and therefore, the eggshell concentration will not reflect the overall level in the organism. Despite these drawbacks, eggshells are commonly used to monitor the exposure of bird populations to elements because a high number of samples may be collected noninvasively from the breeding site (Dauwe et al. 1999; Mora 2003).

Colonial waterbirds (e.g., gulls, terns, herons, and egrets) are convenient species in which to monitor element concentrations in eggshells because they breed at high densities, which allows many samples to be collected (Ayas et al. 2008; Fu et al 2014). Here, we analyzed the intra-clutch and inter-colony variability in the concentrations of 17 trace elements in the eggshells of black-headed gulls, *Chroicocephalus ridibundus*, breeding in five colonies in northern Poland. The studied population is migratory and spends the winter in Western Europe (the Netherlands and Germany; P. Indykiewicz, unpublished data), and it returns to the breeding grounds in March, where it remains for 3–4 weeks. Some individuals even arrive 6 weeks before egg laying (P. Indykiewicz, unpublished ringing data). The black-headed gull is generally omnivorous; while breeding, its diet mainly consists of aquatic and terrestrial invertebrates, plants, and carrion. Black-headed gulls primarily forage within 3 km of the colony (Vernon 1972; Isenmann 1977; Cramp and Simmons 1983), but they also explore areas up to 12–30 km away (Bukaciński et al. 2015). This species has been considered by various researchers as either a capital (Drent and Daan 1980) or income breeder (Thyen and Becker 2006) because many single-brooding birds, including larids, adopt a mixed strategy to gain nutrients for egg production. Some nutrients are endogenous reserves acquired before breeding, whereas others are acquired near the breeding colony (Klaassen et al. 2004; Drent 2006; Stephens et al. 2009; Blight 2011). Considering that the black-headed gulls in this study spent a relatively long time (3–4 weeks, up to 6 weeks) near their colonies before egg laying, we expect that the element levels accumulated in their eggs will mainly reflect local contamination of their food and environment. The populations were easily sampled because black-headed gulls nest colonially.

Our study aimed to (1) determine whether the concentrations of heavy metals and other elements in the eggshells of black-headed gulls varied among the studied sites; (2) compare the proportions of various habitat types around the studied colonies; and (3) investigate the intra-clutch variability in the concentrations of heavy metals and other elements in the eggshells. Considering that the studied colonies were in a landscape dominated by farmland, we expected higher concentrations of Pb, Cd, Mo, Cu, As, and Zn in the eggshells due to the use of fertilizers and manure in agricultural production. Additionally, because females would be unable to quickly renew their Ca resources following depletion by egg laying, we expected that the eggshell concentrations of other alkaline earth elements, especially Sr (Cusack et al. 2003; Lam et al. 2005), would increase with the number of eggs laid.

## Materials and methods

### Study area

**Fig. 1:**
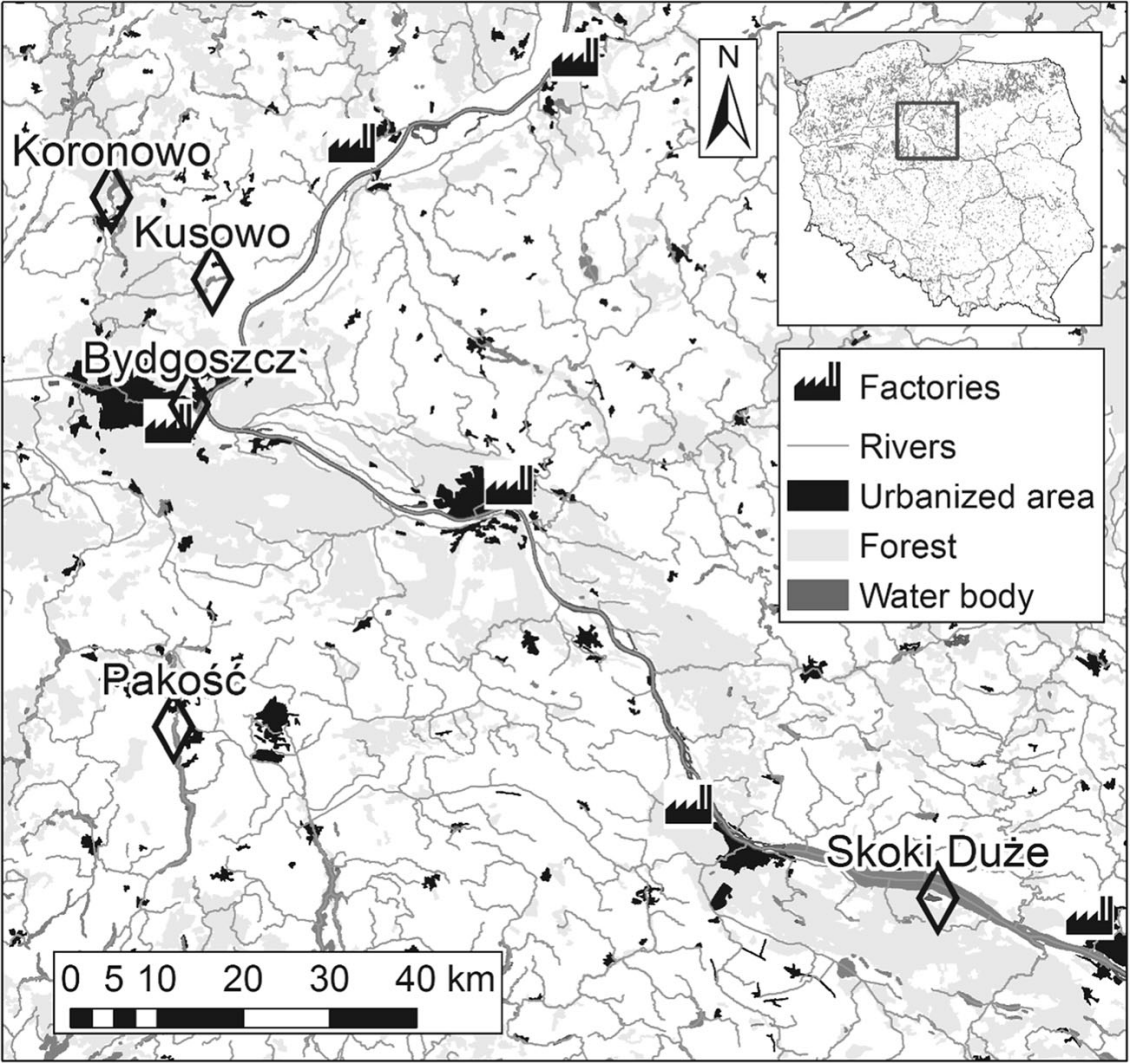
Study area with the locations of all studied black-headed gull colonies (*black diamonds*), the selected land cover types (according to the Corine Land Cover (CLC2006) model; http://www.eea.europa.eu/; EEA Copenhagen, 2012), and the nearest factories (pollution emission sources)

Eggshells were collected from five black-headed gull breeding colonies in the Kuyavian-Pomeranian Voivodeship (northern Poland; Fig. 1): Skoki Duże (52°36.399′, 19°23.643′), Pakość (52°46.973′, 18°05.056′), Kusowo (53°15.015′, 18°08.525′), Koronowo (53°20.069′, 17°57.884′), and Bydgoszcz (53°07.136′, 18°06.318′; the full list of colony characteristics is presented in Table 1).

**Table 1:** Characteristics of the studied black-headed gull colonies

Colony/estimated no. of pairs	Colony characteristic	Local sources of contamination
Skoki Duże/800–1300	Situated on two sandy islets overgrown with grass in a deep, oligotrophic artificial water body in a functioning gravel pit; the colony is surrounded by agricultural areas and a small deciduous woodland.	Fumes emitted from machinery and pollution from conveyor belts and the intensive traffic of heavy dump trucks The nearest factories: a large petrochemical operation in Płock and a chemical plant (nitrogen fertilizers and PVC) in Włocławek are situated 18 and 31 km from the colony, respectively.
Pakość/700–1200	Situated on the steep banks of islets in the mesotrophic Pakoskie Północne Lake; each island supports a metal pillar for a cable car system; the lake serves as a reservoir and is surrounded by vast arable fields.	The intensive use of manure and other fertilizers and the regular application of pesticides around the lake; residues of these contaminants flow from arable land to the lake basin. The nearest factories: chemical plants in Bydgoszcz (polyurethanes and plastics) and Toruń (plastics) are situated 35 and 46 km from the colony, respectively. There is also a large aerospace operation in Bydgoszcz that services and paints aircraft.
Kusowo/900–1200	Situated on a large island (8039 ha) overgrown with lush vegetation, including trees. The island is located in the shallow, mesotrophic Kusowo Lake; extensive monocultures of rapeseed, maize, and other cereals adjoin the lake.	Fertilizer application and intensive pesticide use, especially on rapeseed fields, and the use of manure on fields around the lake; the residues flow to the lake basin. The nearest factories: a paper factory in Świecie and a chemical plant (rubber) in Grudziądz, situated 18 and 19 km from the colony, respectively
Koronowo/130–150	Situated on a small islet in the northern part of Koronowskie Lake, which is an artificial mesotrophic reservoir established in the late 1960s. The lake is used for recreation, and seasonal holiday houses are located near the island.	Pesticides used in the nearby gardening allotments and municipal sewage from large holiday houses and the town of Koronowo (12,000 inhabitants) The nearest factories: a chemical plant in Bydgoszcz (polyurethanes and plastics) and a paper factory in Świecie, situated 27 and 28 km from the colony, respectively
Bydgoszcz/320	Situated in an urban zone on a 50-m^2^ islet in a small artificial bay on the eutrophic Brda River close to the confluence with the Vistula River; factories, service buildings, and a railway line are located nearby. Watersports are popular on the river near the island	Municipal pollution from Bydgoszcz, which has 350,000 inhabitants The nearest factories: chemical plants in Bydgoszcz (polyurethanes and plastics) and Toruń (plastics) are situated 3 and 38 km from the colony, respectively.

Throughout the breeding season, black-headed gulls primarily feed around the water bodies in which their colonies are located, except at Kusowo and Pakość, where they mostly feed in arable fields. At Skoki Duże and Bydgoszcz, the gulls forage in the Vistula River valley. Except for the Skoki Duże colony, garbage dumps are located within approximately 2 km of each monitored colony; these dumps serve as an additional source of food for the black-headed gulls (P. Indykiewicz, unpublished data).

### Field methods

Female gulls laid their eggs between 7 April and 2 May 2015 (Julian days 97–122), and we collected eggshells from randomly selected nests containing three eggs every week. Using a washable marker, we marked each laid egg with a reference number at both ends. Chicks hatched after 23–26 days (Cramp and Simmons 1983; P. Indykiewicz, unpublished data), and we immediately searched for the eggshells within 3–4 m of the nests. We first cleaned the larger chunks of soil and feces from the collected eggshells before placing them in airtight containers (Rotilabo, Roth, Germany) and delivering them to the laboratory.

We analyzed eggshells from 29 three-egg black-headed gull clutches: six from Bydgoszcz, six from Koronowo, six from Kusowo, six from Pakość, and five from Skoki Duże. Three-egg clutches dominated in the studied colonies, constituting 74.8% of all clutches in 2015 (74.9% in Bydgoszcz, 68.7% in Koronowo, 72.4% in Kusowo, 75.0% in Pakość, and 83.2% in Skoki Duże). The mean clutch size (±SD) was 2.86 ± 0.48 eggs/nest (*N* = 187) at Bydgoszcz, 2.76 ± 0.51 eggs/nest (*N* = 131) at Koronowo, 2.85 ± 0.50 eggs/nest (*N* = 145) at Kusowo, 2.87 ± 0.48 eggs/nest (*N* = 124) at Pakość, and 2.97 ± 0.41 eggs/nest (*N* = 161) at Skoki Duże. We examined all of the colonies during laying and hatching to determine the laying dates and the hatching sequence at each nest.

### Analytical procedures

We first removed the inner membrane and then washed the eggshells with deionized water, rinsed them with acetone, and ground them in a ceramic mortar before taking the measurements. To minimize the risk of metal contamination, all glassware and utensils were soaked in an acid bath (5 M HNO_3_) for 24 h, rinsed with demineralized water, and dried under a laminar flow hood before use. Each eggshell was divided into two subsamples and analyzed in duplicate (the mean value per eggshell was used in all calculations). We poured 10 mL of concentrated HNO_3_ (Sigma-Aldrich, Chempur, Poland) over 500 ± 1 mg of eggshell and wet-washed the sample. We mineralized each sample with a microwave digestion system using optical, temperature, and pressure monitoring during acid digestion (Berghof Speedwave, Eningen, Germany) in Teflon vials (type DAP 100). The mineralization scheme was as follows: 15 min from room temperature to 140 °C, 5 min at 140 °C, 5 min from 140 to 170 °C, 15 min at 170 °C, and cooling to room temperature (variable); the pressure did not exceed 12 bar during mineralization. The clear solution obtained after mineralization was cooled to room temperature and transferred to 50-mL flasks, which were filled with demineralized water (ELGA Pure Lab Classic) to the indicated level. We used an iCAP Series 6500 inductively coupled plasma optical emission spectrometer from Thermo Scientific (USA), equipped with a charge injection device, to determine the element concentrations. The spectrometer was controlled by PC-based iTEVA software using the following parameters: RF generator power, 1150 W; RF generator frequency, 27.12 MHz; coolant gas flow rate, 16 L min^−1^; carrier gas flow rate, 0.65 L min^−1^; auxiliary gas flow rate, 0.4 L min^−1^; max integration time, 15 s; pump rate, 50 rpm; viewing configuration, axial; replicates, 3; flush time, 20 s (iCAP 2010). We used the following multi-element stock solutions from Inorganic Ventures as standards:

- Analytic—46: Cu, Fe, Mg, P, K, and Na in 5% HNO_3_, 1000 μg mL^−1^
- Analytic—47: Al, As, Cd, Cr, Pb, Mn, Hg, Ni, Sc, Se, Sr, V, and Zn in 10% HNO_3_, 100 μg mL^−1^
- Analytic—83: Ca, K, Mg, Na, P, and S in 2% HNO_3_, 1000 mg L^−1^
- CGMO1-1: Mo in H_2_O with traces of NH_4_OH, 1000 μg mL^−1^

Based on the mineralization method (dilution of 500 mg of sample in 10 mL of HNO_3_ with a density of 1.51 g cm^−3^), the Hg detection limit was estimated to be 0.058 μg/L (3.72 × 10^−5^ mg kg^−1^).

We ran all of the samples in batches (colony), and each colony included a blank (control) sample. We used a certified reference material, TraceCERT - Periodic table mix 1 for ICP (Fluka Analytical, Sigma-Aldrich), to control the accuracy of the analysis under the existing working conditions. To calculate the recovery percentage, we individually supplied three randomly selected samples with known amounts of the analytical standard and used concentrations of all of the examined elements as positive controls (Table 2). We calculated the mean percentage recoveries of the analyzed elements based on the following equation: Recovery [%] = (*C*_E_/*C*_S_ × 100), where *C*_E_ is the experimental concentration determined from the calibration curve and *C*_S_ is the spiked concentration. All of the concentrations obtained in this study are presented in milligrams per kilogram dry weight.

**Table 2:** Validation of the analytical method used in this study: linearity (the ability of the method to obtain test results proportional to the concentration of the analyte), detection limits and recoveries for the studied elements

Element	Linearity Correlation coefficient *r*	Limit of detection, LOD (μg L^−1^)	Recovery (%)
Al	0.9997	0.021	101
As	0.9995	0.011	99
Ca	0.9985	0.002	105
Cd	0.9999	0.001	97
Cr	0.9997	0.003	97
Cu	0.9999	0.002	103
Fe	0.9998	0.021	96
Hg	0.9996	0.058	97
Mg	0.9953	0.005	104
Mn	0.9998	0.002	96
Mo	0.9996	0.022	98
Ni	0.9999	0.001	97
Pb	0.9999	0.010	98
Sc	0.9997	0.002	99
Se	0.9995	0.012	97
Sr	0.9998	0.003	98
V	0.9999	0.003	97
Zn	0.9998	0.010	102

### Habitat analyses

To identify the main habitat types in the 20-km buffers around the studied colonies (reflecting the range in foraging flights), we extracted data from the Corine Land Cover (CLC2006) model (http://www.eea.europa.eu/, EEA Copenhagen, 2012), which contains land cover information derived from Landsat 7 satellite images. We compared the areas of particular habitat types associated with the studied colonies using a *G* test. In the case of an area around a colony that lacked a certain habitat type (water courses and wetlands), we added 0.1 to all of the values for that colony. We performed spatial analyses (the extraction of particular landscape features and the calculation of the distances between colonies) using ArcMap software, version 10.3.1 (ArcGIS, ESRI, Redlands, CA, USA). We used a Spearman rank correlation to investigate the relationship between the percentage of an area covered by a habitat type and the concentrations of particular elements.

### Statistical analyses

To identify groups of elements with high degrees of association/correlation, we performed the following analyses:

1. Pearson correlation: we determined the correlation strength according to Hinkle et al. (2003), i.e., strong correlation (*r* = |0.90–1.00|), high correlation (*r* = |0.70–0.90|), and moderate correlation (*r* = |0.50–0.70|).
2. Principal component analysis (PCA): we used this technique to reduce the number of variables to a few new ones called factors that represented groups of elements with significantly correlated concentrations.

To investigate intra-clutch differences in the element concentrations, we used models incorporating the laying date (Julian date) and the laying order (the number of the egg in laying sequence) as fixed effects and nest identity (nest ID) as a random effect. First, we applied a full linear mixed model with a random effect (LMM) followed by a general linear model without a random effect (LM). We compared the relative performance of the models by comparing their Akaike information criterion values and a *χ* ^2^ test (Burnham and Anderson 2002). If the LMM performed better than the LM or if there was no significant difference between them, we presented the results of the LMM.

To compare the qualitative and quantitative composition of all trace elements in the eggshells among the studied colonies, we applied the following multivariate methods:

1. Non-metric multidimensional scaling (nMDS): an indirect gradient analysis approach that produces an ordination based on a distance matrix explained by the Bray-Curtis similarity measure (Taguchi and Oono 2005) that was used to visualize the similarity of the element compositions of the colonies
2. Analysis of group similarities (ANOSIM): a procedure based on the Bray-Curtis measure of similarity which was used to test for differences in element compositions among the colonies (Clarke 1993)
3. The similarity percentage breakdown (SIMPER): a procedure that assesses the average percent contribution of particular elements to the dissimilarity between objects in a Bray-Curtis dissimilarity matrix; the significance of the results is determined by ANOSIM (Clarke 1993).

To compare the concentrations of certain elements among the colonies, we used univariate analysis of variance (ANOVA) followed by a post hoc HSD test for unequal *N*.

We determined whether the data sufficiently satisfied the assumptions of the linear model using *Q*-*Q* plots (the quantile expected from normally distributed vs. the quantile from the observed residuals plot). As our data were not normally distributed, we performed log(*x* + 1) (Cd, Cu, Mo, Sc, V) or log (Al, Ca, Cr, Fe, Mg, Mn, Ni, Pb, Se, Sr, Zn) transformations on the data, which resulted in residuals with a normal distribution, before fitting the linear models. We performed multivariate analyses on log(*x* + 1)-transformed data for all elements.

To compare our data with the results from the literature, we present the mean and SD values. We conducted the statistical analyses in STATISTICA 12.0 (StatSoft, Inc. 2014), PAST 3.0 (Hammer et al. 2001), and R software (R Development Core Team 2015).

## Results

### Concentrations of elements

**Fig. 2:**
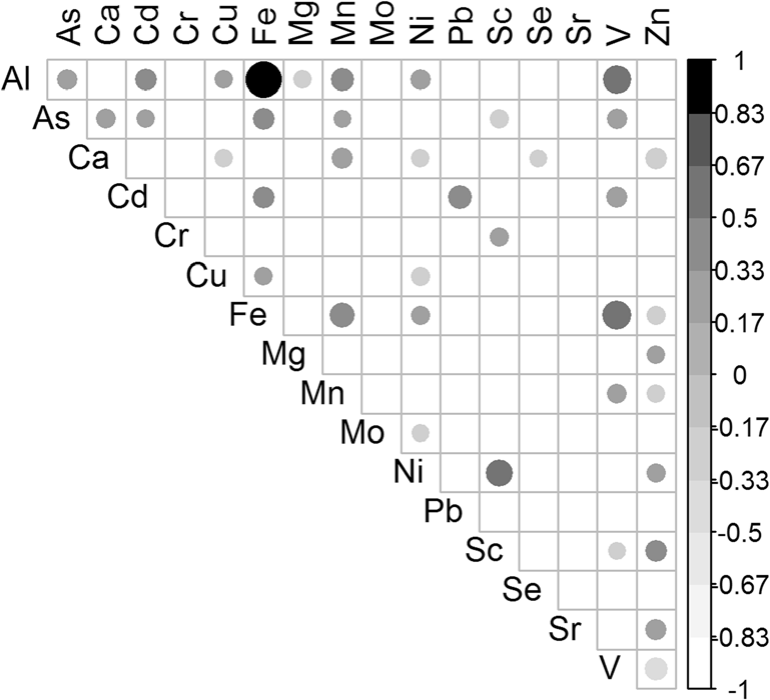
Correlogram—Pearson correlations between all elements detected in the black-headed gull eggshells in Poland. The *size* and *color of the dots* represent the strength of the correlation; only significant relationships are indicated (*P* > 0.05)

The trace element concentrations in the eggshells of the studied black-headed gulls were ordered as follows: Ca > Mg > Sr > Fe > Zn > Al > Cr > Se > Mn > Cu > Pb > As > Ni > Mo > Sc > Cd (Table ES2, Electronic Supplementary Materials). The Al and Fe concentrations were highly significantly positively correlated (Pearson’s correlation: *r* = 0.98, *P* < 0.001; Fig. 2), and moderate positive relationships occurred between the concentrations of V and Fe (*r* = 0.61, *P* < 0.001) and V and Al (*r* = 0.59, *P* < 0.001). The remaining relationships were weaker or not significant (Fig. 2).

PCA revealed that 44.3% of the total variance was explained by the three axes (Table 3). PC1 explained 21.6% of the total variance and was highly positively correlated (*r* = 0.73–0.88) with Al, Fe, and Mn and moderately positively correlated with V (*r* = 0.67; Table 3). PC2 explained 12.2% of the total variance and was highly positively correlated with Sc and Ni (*r* = 0.78–0.83), and PC3 explained 10.5% of the total variance and was moderately positively correlated with Cu, Zn, and Pb (*r* = 0.51–0.53; Table 3).

**Table 3:** Values of the principal component loadings for the studied elements in the eggshells of black-headed gulls

Elements	PC 1	PC 2	PC 3
Al	***0.80***	**0.32**	0.18
As	**0.47**	**−0.31**	**0.30**
Ca	**0.49**	−0.20	**−0.31**
Cd	**0.47**	0.14	**0.43**
Cr	0.00	**0.35**	**−0.48**
Cu	0.03	−0.20	**0.53**
Fe	***0.89***	**0.26**	0.11
Mg	**−0.37**	−0.10	0.18
Mn	***0.73***	**0.23**	−0.15
Mo	0.21	**−0.36**	−0.02
Ni	−0.08	***0.78***	0.16
Pb	0.05	−0.08	**0.51**
Sc	−0.17	***0.83***	−0.03
Se	−0.20	0.12	**0.38**
Sr	−0.17	−0.07	**0.29**
V	**0.68**	−0.12	0.17
Zn	**−0.53**	**0.26**	**0.52**
Eigenvalues	3.7	2.1	1.8
Total variance explained (%)	21.6	12.2	10.5

All significant correlations (*P* > 0.05) are highlighted in bold and highly correlated values (*r* > 0.70) are in italics

### Intra-clutch variation in trace element concentrations

**Fig. 3:**
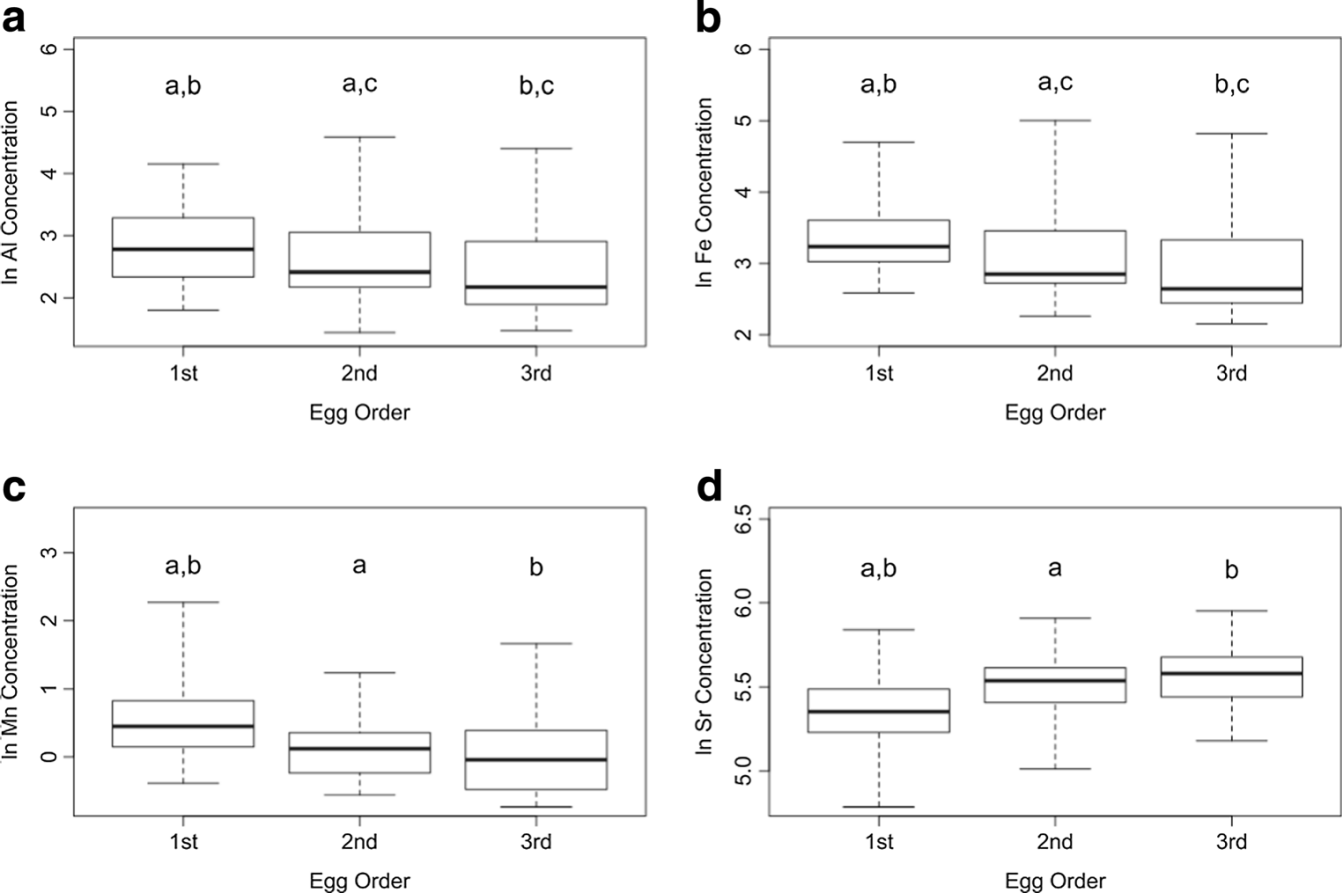
Concentrations (median, *bold horizontal line*; interquartile range, *box*; min–max, *whiskers*) of aluminum (**a**), iron (**b**), manganese (**c**), and strontium (**d**) in consecutively laid black-headed gull eggs. *a*, *b*, *c* are significant differences between particular eggs (post hoc Tukey’s test: *P* < 0.05)

In most of the analyses, we found that the LMM with nest ID as the random factor performed better than the LM without a random factor (Table ES2, Electronic Supplementary Materials). The concentrations of Al and Fe were significantly affected by the egg laying sequence (*P* < 0.001 for both). The Al and Fe concentrations in the first egg were higher than in the second and third eggs (post hoc Tukey’s test: both *P* < 0.001), and the Al and Fe concentrations in the second egg were higher than those in the third egg [Tukey’s test: Al: *P* = 0.007 (Fig. 3a) and Fe: *P* = 0.006 (Fig. 3b)]. The effect of the Julian egg laying day was not significant (*P* = 0.12 for both).

The concentrations of Mn and Sr were significantly affected by the egg laying sequence (Mn: *P* = 0.003 and Sr: *P* < 0.001), and the concentrations were ordered as follows: first egg > scond egg > third egg (Fig. 3c, d). The Mn concentration in the first egg was significantly higher than that in the second (Tukey’s test: Mn: *P* = 0.01 and Sr: *P* < 0.001) and third eggs (Mn: *P* = 0.002 and Sr: *P* < 0.001); the values in the second and third eggs were similar (Mn: *P* = 0.71 and Sr: *P* = 0.13). Julian day did not significantly affect the Mn (*P* = 0.30) and Sr (*P* = 0.97) concentrations.

The concentration of Cu tended to be affected by the Julian date (*P* = 0.057), indicating that the concentration increased with the date (*β* = 0.22), but the egg laying sequence was not significant (*P* = 0.48).

For the remaining elements (As, Ca, Cd, Cu, Cr, Mg, Mo, Ni, Pb, Sc, Se, V, and Zn), neither Julian date nor laying order affected their concentrations in the eggshells (*P* > 0.05).

### Habitat differences among colonies

The areas around the studied colonies mainly consisted of agricultural land and forest, which constituted 56.6 and 32.4%, respectively, of the area in the 10-km buffers around the colonies (Fig. 1). However, the distribution of the agricultural areas, urbanized areas, forests, and water courses around the colonies was uneven (*G* tests for goodness of fit: *P* > 0.05 for all), although the proportions of water bodies and wetlands were similar (*G* test: *P* > 0.40; Table 4). The vicinities of the Pakość and Kusowo colonies were characterized by the highest proportion of agricultural land, i.e., 85.8 and 70.7%, respectively. Only one colony, at Bydgoszcz, was located in a more urbanized area (27.6%; Fig. 1). Aquatic habitats constituted little of the 10-km zones around the colonies (Table 4), except for the Skoki Duże colony where the Włocławek Reservoir (an artificial water body established after the Vistula River was dammed) constituted 11.2% of the surrounding 10-km zone (Table 4 and Fig. 1). The proportions of particular habitats in each area were not significantly correlated with the concentrations of certain elements in the eggshells (Spearman rank correlation: all *P* > 0.15), except for wetland which was highly positively related to the Cu concentration (*r*_s_ = 0.92, *P* = 0.03, *N* = 5).

**Table 4:** Relative contribution (in percent) of the habitats within a 10-km radius around the colonies of black-headed gulls, *Chroicocephalus ridibundus* (according to the Corine Land Cover (CLC2006) model; http://www.eea.europa.eu/; EEA Copenhagen, 2012)

Habitat type	Bydgoszcz	Koronowo	Skoki Duże	Kusowo	Pakość	Total	*G* test: *P*
Agriculture areas	27.6	51.6	47.2	70.7	85.8	56.6	0.004
Urbanized area	20.9	1.6	0.3	1.6	4.2	5.7	0.002
Forested areas	47.9	42.2	40.3	25.3	6.3	32.4	0.0009
Water bodies	0.2	4.5	0.9	0.5	3.7	1.9	0.41
Water courses^a^	3.4	0.1	11.2	1.9	–	3.3	0.046
Wetlands^a^	–	0.02	–	0.16	0.08	0.1	1.00

*G* test: result of a *G* test comparing the proportions of particular habitat types among the studied colonies

^a^After adding 0.1

### Inter-colony differences in element concentrations

**Fig. 4:**
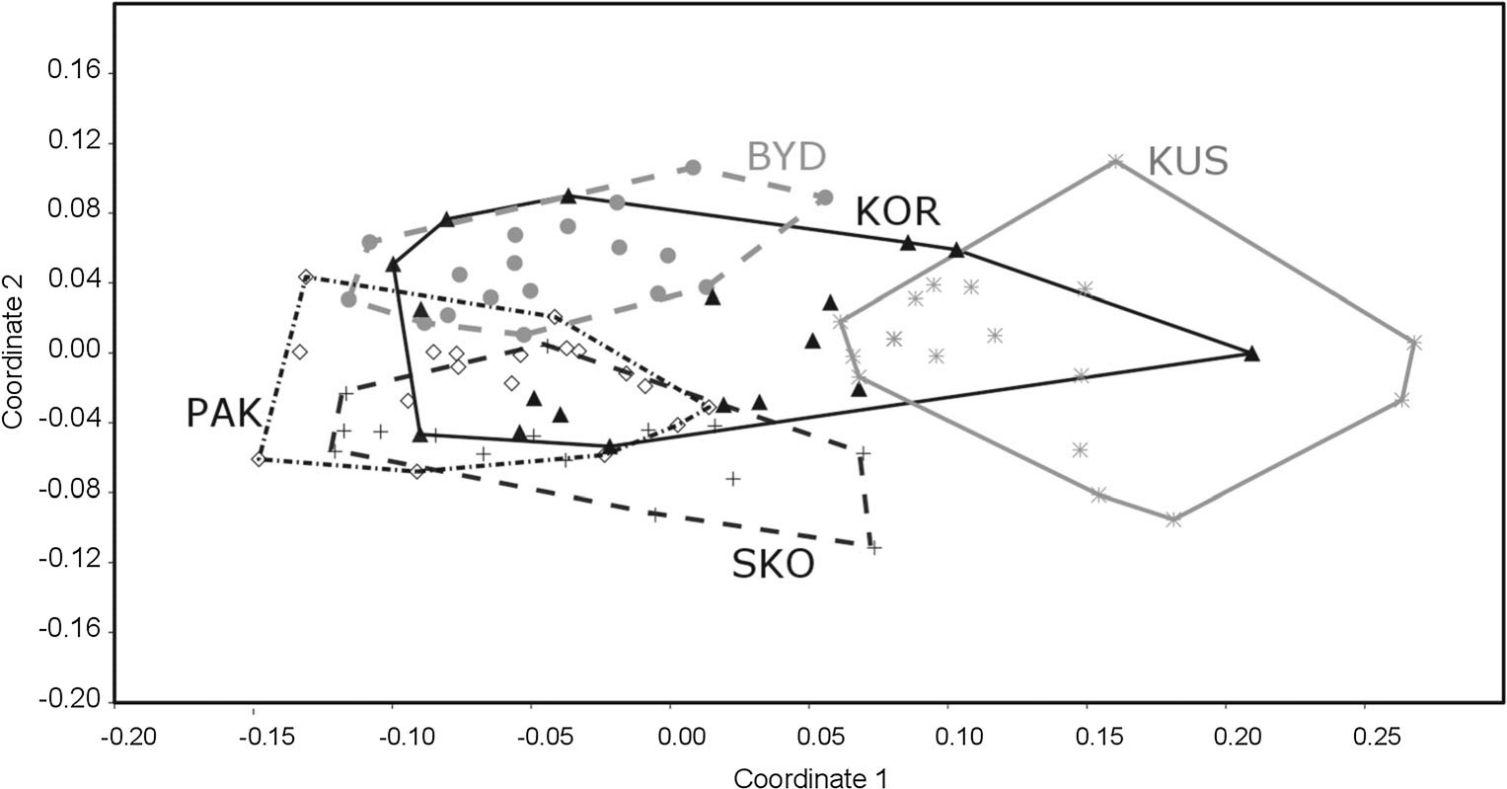
nMDS plots of Bray-Curtis similarities in the elemental concentrations in the eggshells of black-headed gulls breeding in northern Poland. *Convex hulls* contain all samples from one colony. Colony codes: *BYD* Bydgoszcz, *KOR* Koronowo, *KUS* Kusowo, *PAK* Pakość, *SKO* Skoki Duże

Multivariate analyses revealed that the concentrations of all the studied elements significantly differed among the colonies (ANOSIM, similarity measure: Bray-Curtis, *R* = 0.416, *P* = 0.001). The percentage dissimilarity in the elemental concentrations in the eggshells from the different colonies ranged from 4.5 to 7.0 (Table 5), with an overall average value of 5.6; all differences between the colonies were significant (ANOSIM: Bonferroni-corrected *P* < 0.05; Table 4). The nMDS plot of the similarity in the elemental concentrations showed no clear separation among the colonies (Fig. 4), but the eggshells from Kusowo generally clustered apart from the rest of the colonies (Fig. 4). The SIMPER analysis showed that Al, Fe, and Zn made the greatest contributions (17, 15, and 14%, respectively) to the observed pattern of dissimilarity in the elemental concentrations (Table 6).

**Table 5:** Inter-colony dissimilarity (in percent) (SIMPER analyses; dissimilarity measure: Bray-Curtis, *above the diagonal*) and differences (Bonferroni-corrected *P* values, ANOSIM analysis; dissimilarity measure: Bray-Curtis, *below the diagonal*) in the concentrations of elements found in the eggshells of black-headed gulls in four breeding colonies in northern Poland

	Skoki Duże	Koronowo	Bydgoszcz	Pakość	Kusowo
Skoki Duże	–	5.23	5.48	4.48	6.99
Koronowo	0.03	–	5.02	5.03	6.07
Bydgoszcz	0.001	0.002	–	4.54	6.59
Pakość	0.002	0.01	0.002	–	6.97
Kusowo	0.001	0.001	0.001	0.001	–

**Table 6:** Sources of variability in the concentrations of elements [log(*x* + 1)-transformed] (average percentage dissimilarity) in the eggshells of black-headed gulls from the studied colonies according to a SIMPER analysis

Element	Average dissimilarity	Contribution (%)
Al	0.942	16.68
Fe	0.858	15.19
Zn	0.805	14.25
Cr	0.602	10.66
Cu	0.562	9.96
Mn	0.528	9.35
Sr	0.333	5.90

Only elements with a contribution of >5% are presented

Univariate analyses revealed that the concentrations of all elements, except for Mo, Pb, and Se, significantly differed among the colonies (ANOVA: *P* < 0.05; Table 7). We found that the Kusowo colony, which was primarily adjacent to agriculture areas, differed the most from the other colonies (i.e., it showed the highest number of inter-colony differences; HSD post hoc test: *P* < 0.05) in the concentrations of the 11 elements (Table 7). The Zn concentration at the Skoki Duże colony was significantly higher than those at the Koronowo, Bydgoszcz, and Kusowo colonies (Table 7), and the Sc concentration at Koronowo was significantly higher than those at Bydgoszcz, Pakość, and Kusowo. The concentrations of the remaining elements usually differed among any two colonies (Table 7).

**Table 7:** Element concentrations (in milligrams per kilogram) in the eggshells of black-headed gulls breeding in five colonies in northern Poland in 2015

	Skoki Duże (15)		Koronowo (18)		Bydgoszcz (18)		Pakość (18)		Kusowo (18)	
	Mean	SD	Mean	SD	Mean	SD	Mean	SD	Mean	SD
Al	14.8a,b	7.8	18.5c,d,e	13.5	8.0a,c,f	2.8	9.6d,g	2.7	34.8b,e,f,g	21.6
As	0.3a,b	0.2	0.4c	0.2	0.5	0.2	0.5a	0.2	0.6b,c	0.2
Ca^a^	326.1a	7.6	326.7b	11.5	330.4	7.0	330.0	7.8	338.5a,b	14.1
Cd	0.018	0.011	0.015	0.005	0.014	0.014	0.012a	0.005	0.021a	0.007
Cr	1.5a	1.1	3.1a,b,c	1.9	2.3	1.0	1.6b	1.1	1.8c	1.2
Cu	0.5a	0.4	0.6b	0.5	0.6c	0.4	0.7d	0.6	1.4a,b,c,d	1.8
Fe	20.4a	11.6	27.1b	22.3	17.0c	5.8	17.8d	5.5	54.6a,b,c,d	31.9
Mg	2081a	231	1922	154	2018	248	2124b	312	1857a,b	222
Mn	1.1a	0.5	1.3b	0.7	2.2c	2.2	0.8c,d	0.2	2.3a,b,d	0.7
Mo	0.10	0.06	0.10	0.03	0.10	0.04	0.10	0.05	0.10	0.05
Ni	0.30a	0.04	0.30 b	0.03	0.20 c	0.03	0.20	0.03	0.20a,b,c	0.06
Pb	0.5	0.2	0.4	0.1	0.6	0.4	0.7	0.8	0.5	0.3
Sc	0.029a,b	0.008	0.032c,d,e	0.010	0.020c	0.011	0.014a,d	0.007	0.013b,d	0.010
Se	1.72	0.18	1.74	0.35	1.65	0.28	1.71	0.27	1.65	0.31
Sr	300a,b,c	55	220a	33	235b	52	242	36	243c	62
V	0.06a	0.05	0.10b	0.05	0.09c	0.04	0.08d	0.04	0.15a,b,c,d	0.06
Zn	35.5a,b,c,d	9.2	22.8a,e	15.5	13.3b,f	2.72	22.8c,f,g	6.80	11.9d,e,g	3.7

Values with lowercase letters are significant inter-colony differences (*P* < 0.05; HSD post hoc test, ANOVA of log- or log(*x* + 1)-transformed data)

*SD* standard deviation

^a^×10^3^

## Discussion

To our knowledge, this is the first study to investigate the concentrations of trace elements in the eggshells of the black-headed gull in Central European areas dominated by farmland. In contrast, previous studies were performed in highly polluted and urbanized areas of Upper Silesia (southern Poland; Migula et al. 2000).

### Concentrations of the chosen elements

Here, we focus on only some of the elements that are either toxic (Hg, Pb, and Cd) or essential (Cu and Zn) to black-headed gulls.

The Cu levels (in milligrams per kilogram) recorded in our study (0.640) are comparable to those reported for black-tailed gulls, *Larus crassirostris*, in northern Japan (0.535; Agusa et al. 2005), but they are lower than the levels observed in various other species of gulls (1.856–10.20; Morera et al. 1997; Ayas et al. 2008; Fu et al. 2014; Kim and Oh 2014) and considerably higher than in black-headed gulls from Cu-polluted areas in southern Poland (9.64 and 7.98; Migula et al. 2000). Very high dietary levels of copper (Stevenson et al. 1983; Richards 1997) in laying birds will cause a rapid cessation of egg production, whereas feeding laying hens a copper-deficient diet results in a shell membrane with a significantly reduced copper concentration and an abnormal structure (Baumgartner et al. 1978; Richards 1997).

The concentration of Zn in the eggshells (in milligrams per kilogram) from our study (17.9) was intermediate, i.e., lower than those reported for the same species in polluted areas in southern Poland (24.5–29.7; Migula et al. 2000) and in South Korea (52.0; Kim and Oh 2014) but higher than those reported (2.35–9.64) for terns and herons in Hong Kong (Lam et al. 2005). Zn is used in cell division and the development of many organs (including feathers), and therefore, a deficiency in this element may negatively affect embryonic development and decrease hatchability (Blamberg et al. 1960; Vallee and Falchuk 1993; Emsley 2001).

We found no measurable levels of Hg (i.e., <0.058 μg L^−1^ or 3.8 × 10^−5^ mg kg^−1^) in the studied eggshells (Table ES2, Electronic Supplementary Materials). It has been reported that avian diets rich in aquatic organisms favor the accumulation of this element (Horai et al. 2007; Nam et al. 2005); however, the omnivorous diet of the black-headed gulls may have prevented high Hg concentrations from occurring (Kitowski et al. 2015). The fact that we did not detect Hg suggested that the studied birds foraged, probably on plants, in areas with low Hg concentrations prior to breeding. A high level of mercury in the diet was shown by Wiemeyer et al. (1984) to result in fewer eggs and higher embryo mortality, and high concentrations may cause cracked eggs due to reduced eggshell strength or eggshell thinning as well as decreased embryonic growth and embryo deformities (Lundholm 1995; Herring et al 2010; Heinz et al. 2012; Daso et al. 2015).

The mean Cd concentrations (in milligrams per kilogram) recorded in our study (0.015) were lower than those reported for the same species in Korea (0.31; Kim and Oh 2014). In many colonial waterbirds, Cd concentrations are low, usually <0.05 (Agusa et al. 2005; Lam et al. 2005); high Cd levels in eggshells are rare and mainly occur in urbanized or industrial areas, e.g., 5.21–5.25 in the eggshells of black-headed gulls from polluted areas of southern Poland (Migula et al. 2000) and 7.72–9.12 in the eggshells of gulls and herons from eastern China (Fu et al. 2014). Studies suggest that exposure to Cd, even a single dosage, can hamper avian reproduction for a short time, mainly by decreasing egg production and causing eggshell thinning and deformed embryos (Furness 1996; Rahman et al. 2007)

The eggshells of many species of birds reflect Pb contamination in their environment, especially in polluted areas (Dauwe et al. 1999; Fu et al. 2014). Like Cd, Pb can disrupt Ca metabolism by competing with Ca^2+^ and can interfere with the processes that use these ions; this harms embryos and eggshells (Richards and Packard 1996). Furthermore, birds discharge Pb into their feathers and eggshells. The levels (in milligrams per kilogram) of Pb we found in this study (0.47) are low compared to those in the eggshells of black-headed gulls from polluted areas in southern Poland (66.21–66.41; Migula et al. 2000) and in the eggshells of gulls and herons (78.72–91.95) from polluted areas in eastern China (Fu et al. 2014).

### Intra-clutch variation in trace element concentrations

Our analyses revealed a significant effect of the egg laying sequence on the Al, Fe, Mn, and Sr eggshell concentrations. In Audouin’s gull, such an effect has been observed for Mn and Hg (Morera et al. 1997); we likely did not observe an effect on Hg due to the lack of detectable concentrations of this element. The Mn concentrations in the eggshells of consecutive black-headed gull eggs followed the pattern of first egg > second egg > third egg. In the eggshells of Audouin’s gulls, Mn concentrations followed a different sequence (first egg < second egg > third egg; Morera et al. 1997), and these discrepancies might be the result of different temporal variations in the available Mn for both gull species. Audouin’s gulls are mainly piscivorous (Gonzalez-Solis et al. 1997; Morera et al. 1997), while black-headed gulls are omnivorous (Cramp and Simmons 1983). Therefore, the main sources of Mn for black-headed gulls are common water macrophytes, which accumulate large amounts of this element (Samecka-Cymerman and Kempers 2000; Demirezen and Aksoy 2006).

The Fe concentrations in eggshells and egg yolk are determined by the content of this element in the liver and spleen of laying birds (Welch 1992; Richards 1997). In a study of the greater scaup, *Aythya marila*, females exhibited a decreasing hepatic Fe concentration (in milligrams per kilogram) from 3090 during the period without developing ovaries to 2690 during egg laying. Later, during incubation, it recovered to 4930 mg kg^−1^ (Badzinski et al. 2009). These findings may also explain the mechanism underlying the decline in the Fe concentration and the accompanying laying sequence observed in our study.

Increasing Sr concentrations in consecutive eggs (first egg < second egg < third egg) may be explained by the substitution of this element for Ca under Ca-deficient conditions. Given that Ca availability is generally limited for birds during the pre-laying period (Tilgar et al. 1999; Ruuskanen et al. 2014; Espín et al. 2016a, b), as well as the short laying intervals of black-headed gulls (Cramp and Simmons 1983), we expected that egg layers would develop a mechanism to substitute the Ca in eggshells with another element. Sr tends to follow Ca as a trace constituent within organisms during nutrient uptake, internal distribution, and excretion (Blum et al. 2001; Dahl et al. 2001). However, we did not find a statistically significant decrease in the Ca concentrations in consecutive eggs, and therefore, an alternative hypothesis would be required to explain the increasing Sr concentration. Black-headed gull broods are characterized by asynchronous hatching, which creates a hierarchy among the chicks (Eising and Groothuis 2003; Lezalova et al. 2005). For this reason, egg quality varies with the laying sequence, and eggs that are laid first are of a higher quality than those that are laid later (Muller et al 2003; Lezalova et al. 2005). Therefore, embryotoxic (Mora 2003) strontium is allocated to the eggshells of consecutive eggs in larger amounts instead of other alkaline earth metals (Ca and Mg); it also plays a role in protecting the embryo.

We found that the concentration of Cu tended to be affected by the Julian date, indicating an increasing concentration with time that may be explained by a temporal variation in the availability of aquatic food. During the pre-laying period, black-headed gulls forage almost exclusively on the remains of aquatic plants as well as aquatic and terrestrial invertebrates, but only occasionally on fish (Vernon 1972; Cramp and Simmons 1983; P. Indykiewicz, unpublished data). A diet rich in aquatic plants and animals promotes a high accumulation of Cu in the liver (Eisler 1998; Xue et al. 2010; Schummer et al. 2011; Horai et al. 2007), from which it might be relocated to the eggs. This type of food becomes increasingly available to black-headed gulls as the vegetation period progresses. Thus, a positive effect of Julian date on the concentration of Cu in eggshells in our study may be explained by higher Cu availability at the end of the egg laying period compared to the beginning of the season.

### Inter-colony differences

As expected, the concentrations of many elements significantly differed among the studied black-headed gull colonies (Table 4), which suggests that the females breeding in these colonies were mainly feeding near their colonies when their eggs formed.

The Kusowo colony generally clustered in a different position from the rest of the colonies (Fig. 4), which might have resulted from the intensive use of fertilizers, manure, and slurry in the surrounding agricultural region as well as erosion from the intensive monocultures surrounding the lake. The highest concentrations of seven elements (Al, As, Ca, Cu, Fe, Mn, and V) among the studied colonies were observed at this site, and the concentration of Cd, which likely originated from fertilizers, was also highest. The concentrations of Mg, Sr, and Zn in eggshells from Skoki Duże were higher compared to those of other colonies, and this was likely the result of the colony nesting in a functioning gravel pit; soil and parent rock are natural reservoirs of these elements (Kabata-Pendias and Pendias 2010). Alternatively, gulls from this colony may have a high proportion of fish in their diet; fish muscles are characterized by high levels of Na, Fe, K, Ca, Zn, and Mg (Radwan et al. 1990; Łuczyńska et al. 2009). Surprisingly, the Pakość colony was characterized by the highest proportion of agricultural area, and we found the maximal Mg value and the minimal Fe level at this site. At the Bydgoszcz colony, despite being located in the most urbanized area, we did not find very high heavy metal concentrations, but we found a high level of Mn in the eggshells from this colony, which may have been related to water contamination from the industrial waste site from the former “Zachem” chemical plant in Bydgoszcz (Pietrucin 2013). The high level of Sc in the eggshells of gulls breeding at the Koronowo colony may be explained by the birds foraging in the airfield in Bydgoszcz, which is associated with an aircraft service facility. This element is emitted during the processing of alloys and steels for repairing aircraft (Wang et al. 2011), and black-headed gulls were regularly observed foraging in the grassland next to the airfield (P. Indykiewicz, personal observation).

Among the studied elements, the concentration of Zn differed the most among all of the studied colonies, and this may be explained by varying contributions of fish in the different diets (this prey type is rich in Zn; Radwan et al. 1990; Łuczyńska et al. 2009) and/or the intensity of agricultural use (natural and mineral fertilizers serve as an important source of the total annual inputs of Zn into agricultural soils; Nicholson et al. 2003). Inter-colony variation in the Ca and Sr concentrations in eggshells was reflected in the inter-regional variations in their content in the subsurface soil layer (0–5 m; Polish Geological Institute 2005; Fig. ES1). The significant positive correlation between the proportion of wetland areas around the colonies and the concentration of Cu in the eggshells, despite the small sample size, is a good indicator of the availability of Cu-rich aquatic plants and fish (Eisler 1998; Xue et al. 2010) for birds from particular colonies.

Other inter-colony differences in element concentrations may be explained by various factors including differences in local geochemistry, the intensity of agricultural use (various levels of Mg, Zn, and Cd; Nicholson et al. 2003; Aziz et al. 2015; Mazur and Mazur 2016), the varying contributions of fish to the diets (this prey type is rich in Na, Fe, K, Ca, Zn, and Mg; Radwan et al. 1990; Łuczyńska et al. 2009), and the foraging of gulls from some colonies in the Vistula River, which transports multiple pollutants (each year, it transports 28 tons of Zn, 70 tons of Cu, 26.1 tons of Pb, 0.9 tons of Cd, 25.2 tons of Ni, and 200 kg of Hg to the Baltic Sea; Polish Central Statistical Office 2013) and is a considerable source of fish and other aquatic organisms that are contaminated by those elements.

Most of the colonies we studied were situated in agricultural areas, and therefore, mineral fertilizers, especially those from phosphate rocks, served as the main source of Cd and Pb contamination (Charter et al. 1995; Sady and Smolen 2004; Lugon-Moulin et al. 2006; Nziguheba and Smolders 2008). Fertilizers might become a more significant source of contamination in the future as black-headed gulls are adapting to the agricultural landscape; the birds often forage socially in fields like other species that are typical of these agricultural areas, such as rooks. Contrary to our hypothesis, we generally did not observe high concentrations of Cd and Pb (except for Kusowo, which was characterized by a large agricultural area around the colony), but this could be typical for Central Europe (including Poland), where the use of agrochemicals is less intense on small farms, which prevail in the studied area (Chlebicka et al. 2009; Czyzewski and Smedzik 2010; Tryjanowski et al. 2011). The relatively close proximity of the studied colonies to large industrial chemical factories was not reflected in elevated levels of heavy metals. This may indicate the effectiveness of pollution emission reduction systems as confirmed by reports of the Inspectorate of Environment Protection (WIOS w Bydgoszczy 2014; WIOS w Warszawie 2014) and the low concentrations of As, Cr, Zn, Cu, Ni, Pb, and V in the subsurface soil layer (0–5 m) in the studied area (Polish Geological Institute 2005).

## Conclusions

The observed levels of the elements in this study indicate that the environment around the black-headed gull breeding colonies remains in a relatively unpolluted state, which was reflected in the low levels of Cd, Ni, As, Mo, Cu, and Pb as well as the lack of measurable levels of Hg. The work presented here can be treated as a pilot study, and the data can contribute to the planning of more broad-scale monitoring of the local environment that incorporates various tissues and species with different diets.

## Electronic supplementary material

Below is the link to the electronic supplementary material.

ESM 1 (DOC 295 kb)

## References

[CR1] Agusa T, Matsumoto T, Ikemoto T, Anan Y, Kubota R, Yasunaga G, Kunito T, Tanabe S, Ogi H, Shibata Y (2005). Body distribution of trace elements in black‐tailed gulls from Rishiri Island, Japan: age‐dependent accumulation and transfer to feathers and eggs. Environ Toxicol Chem.

[CR2] Ayas Z, Celikkan H, Aksu ML (2008). Lead (Pb) and copper (Cu) concentration in the eggshells of Audouin’s gulls (*Larus audouinii*) in Turkey. Turk J Zool.

[CR3] Aziz T, Maqsood MA, Kanwal S, Hussain S, Ahmed HR, Sabir M, Hakeem KR (2015). Fertilizers and environment: issues and challenges. Crop production and global environmental issues.

[CR4] Badzinski SS, Flint PL, Gorman KB, Petrie SA (2009). Relationships between hepatic trace element concentrations, reproductive status, and body condition of female greater scaup. Environ Pollut.

[CR5] Baumgartner S, Brown DJ, Salevsky E, Leach RM (1978). Copper deficiency in the laying hen. J Nutr.

[CR6] Blamberg DL, Blackwood UB, Supplee WC, Combs GF (1960). Effect of zinc deficiency in hens on hatchability and embryonic development. Exp Biol Med.

[CR7] Blight LK (2011). Egg production in a coastal seabird, the glaucous-winged gull (*Larus glaucescens*), declines during the last century. PloS One.

[CR8] Blum JD, Taliaferro EH, Holmes RT (2001). Determining the sources of calcium for migratory songbirds using stable strontium isotopes. Oecologia.

[CR9] Bukaciński D, Bukacinska M, Zieliński P (2015) Black-headed gull *Chroicocephalus ridibundus*. In: Chylarecki P, Sikora A, Cenian Z, Chodkiewicz T (eds) Monitoring of breeding birds. Methodological guide. Bogucki Wydawnictwo Naukowe, Poznań, pp 266–273 (in Polish)

[CR10] Burger J (1994). Heavy metals in avian eggshells: another excretion method. J Toxicol Environ Health.

[CR11] Burger J (2002). Food chain differences affect heavy metals in bird eggs in Barnegat Bay, New Jersey. Environ Res.

[CR12] Burnham KP, Anderson DR (2002). Model selection and multimodel inference: a practical information-theoretic approach.

[CR13] Central Statistical Office (2013). Statistical yearbook of maritime economy.

[CR14] Charter RA, Tabatabai MA, Schafer JW (1995). Arsenic, molybdenum, selenium, and tungsten contents of fertilizers and phosphate rocks. Commun Soil Sci Plant Anal.

[CR15] Chlebicka A, Fałkowski J, Wołek T (2009). Small farms in Poland—characteristic.

[CR16] Clarke KR (1993). Non-parametric multivariate analysis of changes in community structure. Aust J Ecol.

[CR17] Cramp S, Simmons KEL (1983). Handbook of the birds of Europe, the Middle East and Africa. The birds of the western Palearctic, vol. III. Waders to gulls.

[CR18] Cusack M, Fraser AC, Stachel T (2003). Magnesium and phosphorus distribution in the avian eggshell. Comp Biochem Physiol B.

[CR19] Czyzewski A, Smedzik K (2010). Technical and environmental efficiency of farms in Poland in 2006–2008, according to their types and sizes. Rocz Nauk Roln Ser G.

[CR20] Dahl SG, Allain P, Marie PJ, Mauras Y, Boivin G, Ammann P, Tsouderos Y, Delmas PD, Christiansen C (2001). Incorporation and distribution of strontium in bone. Bone.

[CR21] Daso AP, Okonkwo JO, Jansen R, Brandao JD, Kotze A (2015). Mercury concentrations in eggshells of the southern ground-hornbill (*Bucorvus leadbeateri*) and wattled crane (*Bugeranus carunculatus*) in South Africa. Ecotox Environ Safety.

[CR22] Dauwe T, Bervoets L, Blust R, Pinxten R, Eens M (1999). Are eggshells and egg contents of great and blue tits suitable as indicators of heavy metal pollution?. Belg J Zool.

[CR23] Dauwe T, Janssens E, Bervoets L, Blust R, Eens M (2005). Heavy-metal concentrations in female laying great tits (*Parus major*) and their clutches. Arch Environ Contam Toxicol.

[CR24] Demirezen D, Aksoy A (2006). Common hydrophytes as bioindicators of iron and manganese pollutions. Ecol Indic.

[CR25] Drent RH (2006). The timing of birds’ breeding seasons: the Perrins hypothesis revisited especially for migrants. Ardea.

[CR26] Drent RH, Daan S (1980). The prudent parent—energetic adjustments in avian breeding. Ardea.

[CR27] Eising CM, Groothuis TG (2003). Yolk androgens and begging behaviour in black-headed gull chicks: an experimental field study. Animal Behav.

[CR28] Eisler R (1998). Copper hazards to fish, wildlife, and invertebrates: a synoptic review.

[CR29] Emsley J (2001). Nature’s building blocks. An A–Z guide of the elements.

[CR30] Espín S, Ruiz S, Sánchez-Virosta P, Eeva T (2016a) Effects of calcium supplementation on growth and biochemistry in two passerine species breeding in a Ca-poor and metal-polluted area. Environ Sci Pollut Res 23:9809–9921 10.1007/s11356-016-6219-y 26856860

[CR31] Espín S, Ruiz S, Sánchez-Virosta P, Salminen JP, Eeva T (2016b) Effects of experimental calcium availability and anthropogenic metal pollution on eggshell characteristics and yolk carotenoid and vitamin levels in two passerine birds. Chemosphere 151:189–201 10.1016/j.chemosphere.2016.02.074 26943740

[CR32] Forero MG, González-Solís J, Hobson KA, Donázar JA, Bertellotti M, Blanco G, Bortolotti GR (2005). Stable isotopes reveal trophic segregation by sex and age in the southern giant petrel in two different food webs. Mar Ecol Prog Ser.

[CR33] Fu J, Wang Q, Wang H, Yu H, Zhang X (2014). Monitoring of non-destructive sampling strategies to assess the exposure of avian species in Jiangsu Province, China to heavy metals. Environ Sci Pollut Res.

[CR34] Furness RW, Beyer WN, Heinz GH, Redmon-Norwood AW (1996). Cadmium in birds. Environmental contaminants in wildlife: interpreting tissue concentrations.

[CR35] Gonzalez-Solis J, Oro D, Pedrocchi V, Jover L, Ruiz X (1997). Bias associated with diet samples in Audouin’s gulls. Condor.

[CR36] Hammer Ø, Harper DAT, Ryan PD (2001). PAST: paleontological statistics software package for education and data analysis. Palaeontol Electron.

[CR37] Heinz GH, Hoffman DJ, Klimstra JD, Stebbins KR (2012). A comparison of the teratogenicity of methylmercury and selenomethionine injected into bird eggs. Arch Environ Contam Toxicol.

[CR38] Herring G, Ackerman JT, Eagles‐Smith CA (2010). Embryo malposition as a potential mechanism for mercury‐induced hatching failure in bird eggs. Environ Toxicol Chem.

[CR39] Hinkle DE, Wiersma W, Jurs SG (2003). Applied statistics for the behavioral sciences.

[CR40] Horai S, Watanabe I, Takada H, Iwamizu Y, Hayashi T, Tanabe S, Kuno K (2007). Trace element accumulations in 13 avian species collected from the Kanto area, Japan. Sci Total Environ.

[CR41] iCAP (2010) iCAP 6000 Series ICP-OES spectrometer hardware manual. Cambridge, UK

[CR42] Isenmann P (1977). Données sur la biologie de reproduction de la Mouette rieuse en Camargue. Nos Oiseaux.

[CR43] Kabata-Pendias A, Pendias H (2010). Trace elements in soils and plants.

[CR44] Kim J, Oh JM (2012). Metal levels in livers of waterfowl from Korea. Ecotox Environ Safe.

[CR45] Kim J, Oh JM (2014). Trace element concentrations in eggshells and egg contents of black-tailed gull (*Larus crassirostris*) from Korea. Ecotoxicology.

[CR46] Kitowski I, Kowalski R, Komosa A, Sujak A (2015). Total mercury concentration in the kidneys of birds from Poland. Turk J Zool.

[CR47] Klaassen MA, Baarspul T, Deckers T, van Tienen P (2004). The relationship between carbon isotope ratios of hatchling down and egg yolk in black-headed gulls. J Field Ornithol.

[CR48] Lam JC, Tanabe S, Lam MH, Lam PK (2005). Risk to breeding success of waterbirds by contaminants in Hong Kong: evidence from trace elements in eggs. Environ Pollut.

[CR49] Lezalova R, Tkadlec E, Obornik M, Simek J, Honza M (2005). Should males come first? The relationship between offspring hatching order and sex in the black‐headed gull *Larus ridibundus*. J Avian Biol.

[CR50] Łuczyńska J, Tońska E, Łuczyński M (2009). Essential mineral components in the muscles of six freshwater fish from the Mazurian Great Lakes (northeastern Poland). Arch Pol Fish.

[CR51] Lugon-Moulin N, Ryan L, Donini P, Rossi L (2006). Cadmium content of phosphate fertilizers used for tobacco production. Agron Sustain Dev.

[CR52] Lundholm CE (1995). Effects of methyl mercury at different dose regimes on eggshell formation and some biochemical characteristics of the eggshell gland mucosa of the domestic fowl. Comp Biochem Physiol B.

[CR53] Mazur Z, Mazur T (2016). The influence of long-term fertilization with slurry, manure and NPK on the soil content of trace elements. J Elementol.

[CR54] Migula P, Augustyniak M, Kowalczyk K (2000). Heavy metals, resting metabolism rates and breeding parameters in two populations of black-headed gull *Larus ridibundus* from the industrially polluted areas of Upper Silesia, Poland. Acta Ornithol.

[CR55] Mora MA (2003). Heavy metals and metalloids in egg contents and eggshells of passerine birds from Arizona. Environ Pollut.

[CR56] Morera M, Sanpera C, Crespo S, Jover L, Ruiz X (1997). Inter- and intraclutch variability in heavy metals and selenium levels in Audouin’s gull eggs from the Ebro Delta, Spain. Arch Environ Contam Toxicol.

[CR57] Muller W, Dijkstra C, Groothuis TG (2003). Inter-sexual differences in T-cell-mediated immunity of black-headed gull chicks (*Larus ridibundus*) depend on the hatching order. Behav Ecol Sociobiol.

[CR58] Nam DH, Anan Y, Ikemoto T, Okabe Y, Kim EY, Subramanian A, Saeki K, Tanabe S (2005). Specific accumulation of 20 trace elements in great cormorants (*Phalacrocorax carbo*) from Japan. Environ Pollut.

[CR59] Nicholson FA, Smith SR, Alloway BJ, Carlton-Smith C, Chambers BJ (2003). An inventory of heavy metals inputs to agricultural soils in England and Wales. Sci Total Environ.

[CR60] Nziguheba G, Smolders E (2008). Inputs of trace elements in agricultural soils via phosphate fertilizers in European countries. Sci Total Environ.

[CR61] Pietrucin D (2013). Water environment pollution in the area of “Zielona” industrial waste site in the “Zachem” chemical plant, Bydgoszcz (Poland). Biuletyn Państwowego Instytutu Geologicznego.

[CR62] Polish Geological Institute (2005) Central geological database. http://www.pgi.gov.pl/. Accessed 05 November 2015

[CR63] R Core Team (2015). R: a language and environment for statistical computing.

[CR64] Radwan S, Kowalik W, Kornijow R (1990). Accumulation of heavy metals in a lake ecosystem. Sci Total Environ.

[CR65] Rahman MS, Sasanami T, Mori M (2007). Effects of cadmium administration on reproductive performance of Japanese quail (*Coturnix japonica*). J Poultry Sci.

[CR66] Raygoza-Viera JR, Ruiz-Fernández AC, Ruelas-Inzunza JR, Páez-Osuna F (2013). The use of blood in *Anas clypeata* as an efficient and non-lethal method for the biomonitoring of mercury. Bull Environ Contam Toxicol.

[CR67] Reynolds SJ (1997) Uptake of Ingested Calcium during Egg Production in the Zebra Finch (Taeniopygia guttata). The Auk 114:562–569

[CR68] Richards MP (1997). Trace mineral metabolism in the avian embryo. Poultry Sci.

[CR69] Richards MP, Packard MJ (1996). Mineral metabolism in avian embryos. Poul Avian Biol Rev.

[CR70] Ruuskanen S, Laaksonen T, Morales J, Moreno J, Mateo R, Belskii E, Bushuev A, Jarvinen A, Kerimov A, Krams I, Morosinotto C, Mand R, Oreel M, Qvarnstrom A, Slater F, Tiglar V, Visser ME, Winkel W, Zang H, Eeva T (2014). Large-scale geographical variation in eggshell metal and calcium content in a passerine bird (*Ficedula hypoleuca*). Environ Sci Pollut Res.

[CR71] Sady W, Smolen S (2004) The impact of soil–fertilizer factors on the accumulation of heavy metals in plants. 10th National Scientific Symposium on the Effects of Using Fertilizer in Garden Farming, Cracow, pp 269–277 (in Polish)

[CR72] Samecka-Cymerman A, Kempers AJ (2000). Bioindication of heavy metals with aquatic macrophytes: the case of a stream polluted with power plant sewages in Poland. J Toxicol Environ Health A.

[CR73] Schummer ML, Petrie SA, Badzinski SS, Deming M, Chen YW, Belzile N (2011). Elemental contaminants in livers of mute swans on Lakes Erie and St. Clair. Arch Environ Contam Toxicol.

[CR74] Skoric S, Visnjić-Jeftic Z, Jaric I, Djikanovic V, Mickovic B, Nikcevic M, Lenhardt M (2012). Accumulation of 20 elements in great cormorant (*Phalacrocorax carbo*) and its main prey, common carp (*Cyprinus carpio*) and Prussian carp (*Carassius gibelio*). Ecotox Environ Safety.

[CR75] StatSoft Inc. (2014) Statistica (data analysis software system), version 12. www.statsoft.com

[CR76] Stephens PA, Boyd IL, McNamara JM, Houston AI (2009) Capital breeding and income breeding: their meaning, measurement, and worth. Ecol 90:2057–2067 10.1890/08-1369.1 19739368

[CR77] Stevenson MH, Pearce J, Jackson NJ (1983). The effects of dietary intake and of dietary concentration of copper sulphate on the laying domestic fowl: effects on laying performance and tissue mineral contents. Br Poult Sci.

[CR78] Taguchi Y-H, Oono Y (2005). Relational patterns of gene expression via non-metric multidimensional scaling analysis. Bioinformatics.

[CR79] Thyen S, Becker PH (2006). Effects of individual life-history traits and weather on reproductive output of black-headed gulls *Larus ridibundus* breeding in the Wadden Sea, 1991–97. Bird Study.

[CR80] Tilgar V, Mänd R, Leivits A (1999). Effect of calcium availability and habitat quality on reproduction in pied flycatcher *Ficedula hypoleuca* and great tit *Parus major*. J Avian Biol.

[CR81] Tryjanowski P, Hartel T, Baldi A, Szymanski P, Tobolka M, Herzon I, Goławski A, Konvicka M, Hromada M, Jerzak L, Kujawa K, Lenda M, Orłowski G, Panek M, Skórka P, Sparks TH, Tworek S, Wuczyński A, Żmihorski M (2011). Conservation of farmland birds faces different challenges in Western and Central-Eastern Europe. Acta Ornithol.

[CR82] Vallee BL, Falchuk KH (1993). The biochemical basis of zinc physiology. Physiol Rev.

[CR83] Vernon JDR (1972). Feeding habitats and food of the black-headed and common gulls. Bird Study.

[CR84] Wang W, Pranolo Y, Cheng CY (2011). Metallurgical processes for scandium recovery from various resources: a review. Hydrometallurgy.

[CR85] Welch S (1992) Transferrin. The iron carrier. CRC, Boca Raton

[CR86] Wiemeyer SN, Lamont TG, Bunck CM, Sindelar CR, Gramlich FJ, Fraser JD, Byrd MA (1984). Organochlorine pesticide polychlorobiphenyl and mercury residues in bald eagle eggs (1969–1979) and their relationships to shell thinning and reproduction. Arch Environ Contam Toxicol.

[CR87] Xue P, Li G, Liu W, Yan C (2010). Copper uptake and translocation in a submerged aquatic plant *Hydrilla verticillata* (L.f.) Royle. Chemosphere.

