# Supplementary material for: Intra-clutch and inter-colony variability in element concentrations in eggshells of the black-headed gull, Chroicocephalus ridibundus, in northern Poland
Source: Environ Sci Pollut Res Int. 2017 Mar 8;24(11):10341–53. doi: 10.1007/s11356-017-8635-z (PMC5389991; doi:10.1007/s11356-017-8635-z)
Supplement: Supplementary file 1 — (DOC 295 kb) [file 11356_2017_8635_MOESM1_ESM.doc]

**Electronic Supplementary Material**

Intra-clutch and inter colony variability in elements concentrations in the black-headed gull *Chroicocephalus ridibundus* eggshells from N Poland

Ignacy Kitowski, Piotr Indykiewicz, Dariusz Wiącek, Dariusz Jakubas

**Table ES1** The relative performance of the models describing intra-clutch variability in concentrations of elements using models with laying date (Julian date) and laying order (number of egg in laying sequence) as fixed effects, and nest identity (nest ID) as a random effect (LMM Model – linear mixed model with a random effect, LM Model - general linear model without a random effect) according to their Akaike’s formation Criterion (AIC).

| Element | AIC LMM Model | AIC LM Model | Models comparison (χ^2^ test) *P* values |
| --- | --- | --- | --- |
| Al | 103.29 | 171.71 | <0.001 |
| As | -24.42 | -23.32 | 0.078 |
| Ca | -350.19 | -344.42 | 0.005 |
| Cd | -562.92 | -564.41 | 0.476 |
| Cr | 176.74 | 175.47 | 0.392 |
| Cu | 77.77 | 89.00 | <0.001 |
| Fe | 98.58 | 154.03 | <0.001 |
| Mg | -125.18 | -113.08 | <0.001 |
| Mn | 138.20 | 153.28 | <0.001 |
| Mo | -299.80 | -300.78 | 0.313 |
| Ni | -49.62 | -32.25 | <0.001 |
| Pb | 133.06 | 135.58 | 0.034 |
| Sc | -536.75 | -521.20 | <0.001 |
| Se | -59.83 | -59.53 | 0.030 |
| Sr | -62.56 | -17.22 | <0.001 |
| V | -273.95 | -268.19 | 0.005 |
| Zn | 94.55 | 146.36 | <0.001 |

**Table ES2** Concentrations [geometric mean (GM) and standard deviation (±SD)] of trace elements [mg·kg^-1^] in eggshells of black-headed gulls *Chroicocephalus ridibundus* breeding in N Poland. *N* - number of the studied eggshells. Elements with the significant differences in element concentrations according to the laying sequence are bolded

|  | Elements | **Al** | As | Ca* | Cd | Cr | Cu | **Fe** | Mg | **Mn** | Mo | Ni | Pb | Sc | Se | **Sr** | V | Zn |
| --- | --- | --- | --- | --- | --- | --- | --- | --- | --- | --- | --- | --- | --- | --- | --- | --- | --- | --- |
| All  eggs | *N* | 87 | 84 | 87 | 83 | 87 | 71 | 87 | 87 | 87 | 86 | 87 | 87 | 80 | 87 | 87 | 83 | 87 |
|  | GM | 13.45 | 0.46 | 3303.0 | 0.015 | 1.71 | 0.64 | 22.43 | 1982.83 | 1.27 | 0.080 | 0.24 | 0.47 | 0.020 | 1.67 | 240.16 | 0.080 | 17.93 |
|  | SD | 15.40 | 0.18 | 1080 | 0.01 | 1.41 | 0.99 | 23.23 | 253.88 | 1.26 | 0.05 | 0.04 | 0.44 | 0.01 | 0.28 | 54.12 | 0.05 | 11.94 |
| 1^st^ eggs | *N* | 29 | 28 | 29 | 29 | 29 | 21 | 29 | 29 | 29 | 29 | 29 | 29 | 26 | 29 | 29 | 28 | 29 |
|  | GM | 17.21 | 0.46 | 3316. 0 | 0.016 | 1.75 | 0.57 | 28.66 | 1956.36 | 1.66 | 0.09 | 0.24 | 0.46 | 0.02 | 1.65 | 214.45 | 0.09 | 18.15 |
|  | SD | 13.13 | 0.21 | 1077 | 0.010 | 1.12 | 1.15 | 20.42 | 228.25 | 1.64 | 0.06 | 0.05 | 0.35 | 0.01 | 0.27 | 48.12 | 0.05 | 13.16 |
| 2^nd^ eggs | *N* | 29 | 28 | 29 | 28 | 29 | 25 | 29 | 29 | 29 | 28 | 29 | 29 | 28 | 29 | 29 | 27 | 29 |
|  | GM | 13.01 | 0.45 | 3283.6 | 0.014 | 1.87 | 0.64 | 21.79 | 2008.50 | 1.18 | 0.08 | 0.24 | 0.44 | 0.02 | 1.69 | 246.61 | 0.08 | 18.42 |
|  | SD | 17.39 | 0.19 | 1166.0 | 0.006 | 1.77 | 0.94 | 25.99 | 270.93 | 0.79 | 0.04 | 0.04 | 0.58 | 0.01 | 0.25 | 50.55 | 0.05 | 11.08 |
| 3^rd^ eggs | *N* | 29 | 28 | 29 | 26 | 29 | 25 | 29 | 29 | 29 | 29 | 29 | 29 | 26 | 29 | 29 | 28 | 29 |
|  | GM | 10.87 | 0.46 | 3309.6 | 0.014 | 1.53 | 0.70 | 18.07 | 1983.98 | 1.05 | 0.08 | 0.23 | 0.52 | 0.02 | 1.67 | 261.91 | 0.09 | 17.23 |
|  | SD | 15.34 | 0.14 | 1002.9 | 0.010 | 1.26 | 0.93 | 22.72 | 266.16 | 1.12 | 0.04 | 0.04 | 0.35 | 0.01 | 0.33 | 53.89 | 0.06 | 11.87 |

* - [mg·g^-1^]


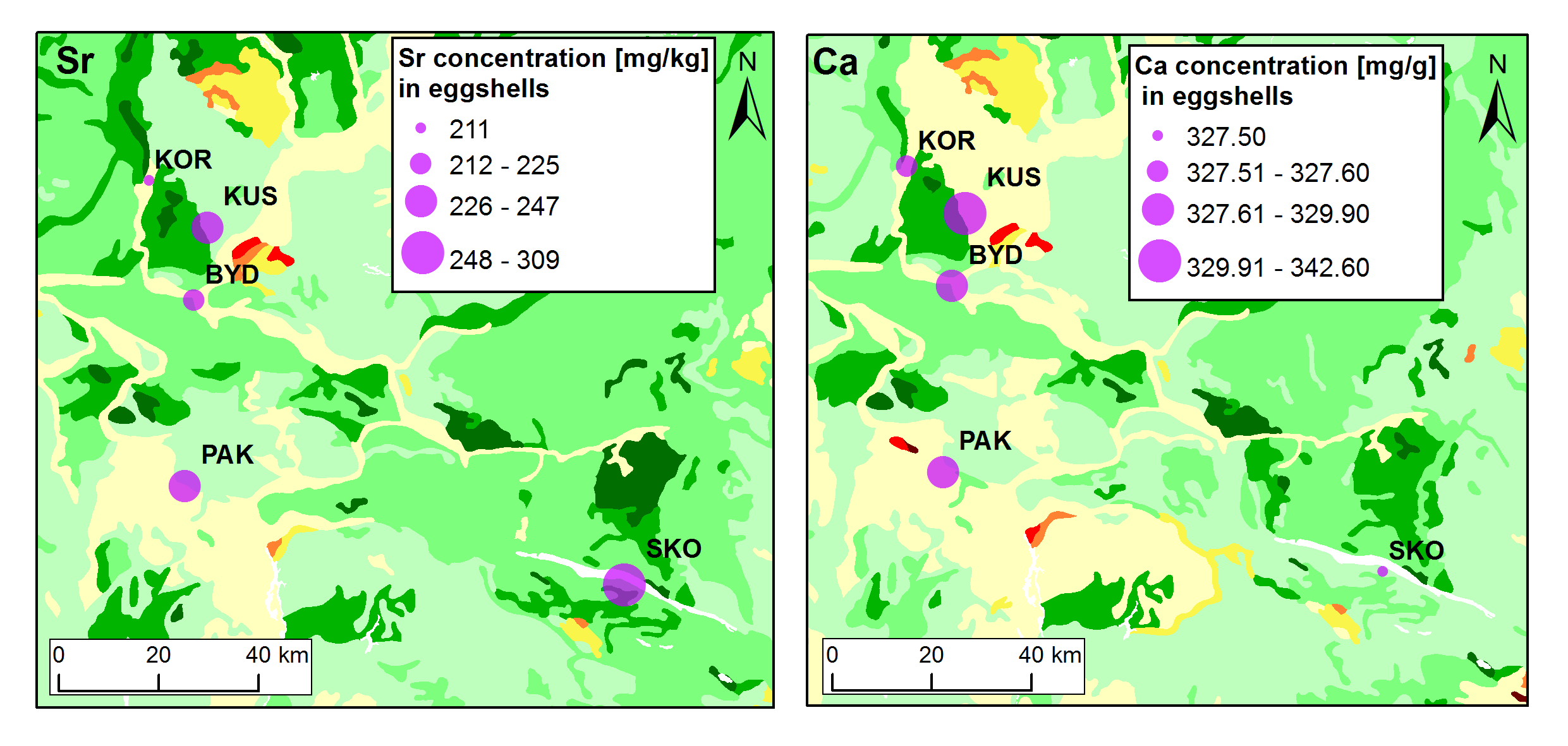


**Fig. ES1** Strontium (Sr) and calcium (Ca) concentration in eggshells of black-headed gulls from the studied colonies (*violet circles*) and in subsurface soil layer (0-5 m) in the studied area (background, yellow and red colours mean higher concentrations; Polish Geological Institute 2005). Colony codes: BYD – Bydgoszcz, KOR - Koronowo, KUS – Kusowo, PAK - Pakość, SKO – Skoki Duże.

**References**

Polish Geological Institute (2005) Central Geological Database. http://www.pgi.gov.pl/. Accessed on 2015.11.05
